# The *anx/anx* Mouse – A Valuable Resource in Anorexia Nervosa Research

**DOI:** 10.3389/fnins.2019.00059

**Published:** 2019-02-05

**Authors:** Ida A. K. Nilsson

**Affiliations:** ^1^Department of Molecular Medicine and Surgery, Karolinska Institutet, Stockholm, Sweden; ^2^Center for Molecular Medicine, Karolinska University Hospital, Stockholm, Sweden; ^3^Centre for Eating Disorders Innovation, Karolinska Institutet, Stockholm, Sweden

**Keywords:** hypothalamus, anorexia, inflammation, neurodegeneration, neuropeptide, AGRP, microglia

## Abstract

Animal models are invaluable resources in research concerning the neurobiology of anorexia nervosa (AN), to a large extent since valid clinical samples are rare. None of the existing models can capture all aspects of AN but they are able to mirror the core features of the disorder e.g., elective starvation, emaciation and premature death. The anorectic *anx/anx* mouse is of particular value for the understanding of the abnormal response to negative energy balance seen in AN. These mice appear normal at birth but gradually develops starvation and emaciation despite full access to food, and die prematurely around three weeks of age. Several changes in hypothalamic neuropeptidergic and -transmitter systems involved in regulating food intake and metabolism have been documented in the *anx/anx* mouse. These changes are accompanied by signs of inflammation and degeneration in the same hypothalamic regions; including activation of microglia cells and expression of major histocompatibility complex I by microglia and selective neuronal populations. These aberrances are likely related to the dysfunction of complex I (CI) in the oxidative phosphorylation system of the mitochondria, and subsequent increased oxidative stress, which also has been revealed in the hypothalamus of these mice. Interestingly, a similar CI dysfunction has been shown in leukocytes from patients with AN. In addition, a higher expression of the *Neurotrophic Receptor Tyrosine Kinase 3* gene has been shown in the *anx/anx* hypothalamus. This agrees with AN being associated with specific variants of the genes for brain derived neurotrophic factor and Neurotrophic Receptor Tyrosine Kinase 2. The *anx/anx* mouse is also glucose intolerant and display pancreatic dysfunction related to increased levels of circulating free fatty acids (FFA) and pancreatic inflammation. An increased incidence of eating disorders has been reported for young diabetic women, and as well has increased levels of circulating FFAs in AN. Also similar to individuals with AN, the *anx/anx* mouse has reduced leptin and increased cholesterol levels in serum. Thus, the *anx/anx* mouse shares several characteristics with patients with AN, including emaciation, starvation, premature death, diabetic features, increased FFA and low leptin, and is therefore a unique resource in research on the (neuro)biology of AN.

## Introduction – Anorexia Nervosa

Anorexia nervosa (AN) is a complex psychiatric disorder affecting around 1% of females and 0.1% of males, of which as many as 10% die as a result of the disorder (Bulik et al., 2006; Keski-Rahkonen et al., 2007; Papadopoulos et al., 2009). The diagnostic criteria, according to the Diagnostic and statistical manual of mental disorders (DSMV), include persistent food intake restriction leading to significantly low body weight, combined with persistent behaviors that interfere with weight gain, and body image distortion (Schaumberg et al., 2017). One central and yet unexplained part of AN is the contradictory response to negative energy balance and the inability to ingest adequate energy, leading to severe underweight. It is indeed paradoxical that while most individuals quickly regain the weight lost from dieting (Pietilainen et al., 2012), individuals with AN stay in an emaciated state commonly for many years, some even until death. It has been speculated that hunger signals are diminished or even absent in individuals with AN, and that satiety signals on the other hand are exaggerated (DeBoer, 2011; Oberndorfer et al., 2013). Supporting this hypothesis, a genome wide association study (GWAS), as well as genetic correlation data, indicate that individuals with AN are genetically predisposed to a lower body weight set point (Duncan et al., 2017; Hinney et al., 2017). However, in order to understand the complex biology of AN, in particular the illogical response to starvation and underweight, we need to learn more about the neurobiological pathways and molecular mechanisms that are associated with severe dysregulation of food intake. This is something that is technically difficult and to some extent impossible to do in humans, since post-mortem tissues rarely are available. On the other hand, animal models cannot capture all aspects of AN but they are able to mirror the core features of the disorder e.g., elective starvation, emaciation and premature death (Siegfried et al., 2003). Animal models have therefore proved to be invaluable resources for researchers in the field. One such model is the *anx/anx* mouse.

## The *anx/anx* Mouse

**FIGURE 1:**
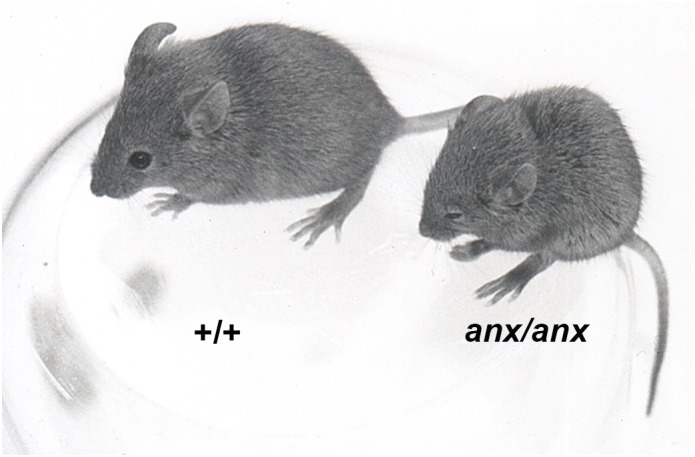
An *anx/anx* mouse and wildtype (+/+) littermate, age 17 days.

The homozygous *anx*-mouse appears normal at birth, meaning that it is indistinguishable from their homozygous and heterozygous wildtype (wt) siblings. However, during the first postnatal weeks they gradually develop the core symptoms of AN; starvation and emaciation (Figure 1). The *anx/anx* mouse dies prematurely around 3 weeks of age, and by then weigh around half as much as their siblings. They are able to eat, but despite full access to milk from the mother, eat significantly less already from postnatal day (P) 5. Worth to note is that the diurnal patterns in food intake seen in their healthy siblings are mirrored in the *anx/anx* mouse, even though the amount ingested is significantly smaller (Maltais et al., 1984). Neurological/behavioral deviations such as head weaving, hyperactivity, body tremors and uncoordinated gait, were described in the original paper by Maltais et al. (1984). When corrected for body weight, brain and thymus weights are increased compared to their healthy siblings, both at P5 and P15, while the weight of spleen is reduced (Maltais et al., 1984). See Table 1 for a summary of the aberrances in the *anx/anx* mouse discussed here and below.

**Table 1:** Main characteristics of the *anx/anx* mouse.

Aberrances of the *anx/anx* mouse	Reference
**Major phenotypes:** reduced food intake, emaciation and premature death.	Maltais et al., 1984
**Organ changes:** increased weight of thymus and brain, and reduced weight of spleen.	Maltais et al., 1984
**Behavioral/Neurological phenotypes:** head weaving, tremor, hyperactivity and uncoordinated gait.	Maltais et al., 1984
**Hypothalamic neuropeptidergic/-transmitter and molecular aberrances:** *- AGRP/NPY*: increased number of AGRP/NPY-immunopositive cell bodies in Arc, reduced number of immunopositive projections. N.C /reduced mRNA expression of AGRP and NPY in Arc. *- POMC/CART*: Reduced number of CART-immunopositive cell bodies in Arc, DMH, LHA, reduced number of immunopositive projections in Arc. Reduced number of Y1-immunopositive cell bodies and projections. Reduced POMC mRNA in Arc. - Increased hypothalamic expression of *Ntrk3*.	Broberger et al., 1998; Nilsson et al., 2008 Jahng et al., 1998; Fetissov et al., 2005 Johansen et al., 2000 Broberger et al., 1999; Nilsson et al., 2011 Mercader et al., 2008b
**Hypothalamic inflammation**, e.g., microglia activation and expression of MHC class I by hypothalamic microglia.	Lachuer et al., 2005; Mercader et al., 2008a; Nilsson et al., 2008
**Hypothalamic degeneration**, e.g., expression of MHC class I by Arc neurons, microglia-associated cell death, increased TUNEL labeling in Arc.	Nilsson et al., 2011
**Mitochondrial dysfunction**, e.g., down regulation of *Ndufaf1* and reduced capacity of CI.	Lindfors et al., 2011
**Neurotransmitter changes in other parts of the brain:** - Increased apoptosis and proliferation in hippocampus. - Serotonergic hyperinnervation of hippocampus, striatum, cortex and cerebellum. - Altered dopaminergic neurotransmission.	Kim et al., 2001 Son et al., 1994 Johansen et al., 2001
**Pancreatic aberrances**, e.g., glucose intolerance, reduced insulin release and inflammation.	Lindfors et al., 2015
**Reduced hypothalamic metabolism**, e.g., reduced glucose uptake, lactate and activation of AMPK, and increased PCr.	Bergstrom et al., 2017
**Changes in serum metabolites**, i.e., reduced leptin and increased FFA.	Johansen et al., 2000; Lindfors et al., 2015


*AGRP, agouti-gene related protein; AMPK, AMP-activated kinase; Arc, the Arcuate nucleus; CI, complex I of the oxidative phosphorylation system; CART, cocaine and amphetamine-regulated transcript; DMH, the dorsomedial hypothalamic nucleus; FFA, free fatty acids; LHA, the lateral hypothalamic area; MHC class I, major histocompatibility complex I; N.C, no change; *Ndufaf1*, The NADH dehydrogenase (ubiquinone) 1a-subcomplex gene; NPY, neuropeptide Y; *Ntrk3*, neurotrophic receptor kinase 3 gene; PCr, phosphocreatine; POMC, pro-opiomelanocortin; TUNEL, terminal dUTP nick end labeling; Y1, neuropeptide Y receptor 1.*

### The *anx* Mutation

The *anx* mutation arose spontaneously at the Jackson laboratory in Bar Harbor, Maine, already in 1976 in the F2 generation of a cross between DW/J and an inbred strain, the latter was derived from a cross between M.m.poschiavinus and an inbred Swiss strain. The male *anx* carrier was crossed to a female B6C3H-a/a F1 mouse, and the mutation has since then been conserved on this background (Maltais et al., 1984). We have mapped the mutation to a 0.2 cM interval residing between the markers D2Mit133 and Jojo5 chromosome 2 (Chr 2: bp 118, 889, 896–120, 175, 108^1^) (Lindfors et al., 2011). So far, no sequencing attempts have been able to show any unique sequence alteration. However, one needs to keep in mind that the background of the *anx/anx* mouse includes five different strains (see above) which makes *de novo* assembly difficult. The lack of unique finding could also mean that the mutation is located in a regulatory element outside the interval. The NADH dehydrogenase (ubiquinone) 1a-subcomplex (*Ndufaf1*) gene, shown to be closely associated with several of the *anx/anx* phenotypes, is however, located in the short interval of the mutation (see section on mitochondrial dysfunction below) (Lindfors et al., 2011). *Ndufaf1* is an assembly factor for complex I (CI) in the mitochondrial oxidative phosphorylation system (OXPHOS) (Vogel et al., 2005). In addition, work by Kim et al. (2017) identified a point mutation in *Tyro3* which they conclude is not the *anx*-mutation but a strain specific modifier of *anx*-phenotypes (Kim et al., 2017). Thus, despite that the *anx/anx* mouse model recently turned 40 years, the mutation is still unknown. Hopefully modern techniques within e.g., sequencing will be able to shed light on this mystery.

### Neurochemistry of the *anx/anx* Mouse

Several changes in neuropeptidergic and -transmitter systems, in particular systems in the hypothalamus known to regulate food intake and metabolism (energy homeostasis), have been documented in the *anx/anx* brain (Broberger et al., 1997, 1999; Johansen et al., 2000, 2003; Nilsson et al., 2013). A part of the hypothalamus, called the Arcuate nucleus (Arc), is of particular importance concerning energy homeostasis. The Arc harbors among others a neuronal population co-expressing two orexigenic neuropeptides; agouti-gene related protein (AGRP) and neuropeptide Y (NPY), and a neuronal population co-expressing the anorexigenic peptide/precursor; pro-opiomelanocortin (POMC) and cocaine and amphetamine-regulated transcript (CART) (Chronwall, 1985; Cone et al., 2001; Schwartz, 2001). Aberrances have been documented in both these neuronal populations in the *anx/anx* mouse. Immunohistochemistry revealed increased number of NPY and AGRP immunopositive cell bodies within the Arc, combined with a reduction in AGRP/NPY immunopositive projections in the hypothalamic and extra-hypothalamic target areas of these neurons (Broberger et al., 1997, 1998; Fetissov et al., 2005; Nilsson et al., 2008). *In situ* hybridization studies have with regard to these neuropeptides been inconsistent, which most likely is attributed to overexposure of the labeled glass slides in the earlier studies. Thus, while initial studies documented no change in mRNA levels of NPY in the Arc of the *anx/anx* mouse (Broberger et al., 1997; Jahng et al., 1998), a later study showed increased mRNA for both NPY and AGRP in the *anx/anx* Arc (Fetissov et al., 2005). With regard to the POMC/CART population, significantly decreased levels of CART mRNA, as well as CART immunopositive cell bodies and fibers in Arc have been shown in the *anx/anx* hypothalamus. Also, a lower number of detectable CART-expressing cells in the dorsomedial hypothalamic nucleus/lateral hypothalamic area is seen (Johansen et al., 2000). *In situ* hybridization demonstrated decreased numbers of POMC-expressing neurons in the *anx/anx* Arc (Broberger et al., 1999). Using the neuropeptide Y receptor 1 (Y1) which outlines the soma and dendrites of POMC/CART neurons (Zhang et al., 1994; Kopp et al., 2002), markedly reduced immunoreactivity in Arc and the paraventricular nucleus of hypothalamus was revealed (Broberger et al., 1999; Nilsson et al., 2011). Clinically, genetic variants of AGRP have been associated with AN (Dardennes et al., 2007) or with lowest BMI during AN illness (Yilmaz et al., 2014). Increased plasma levels of the peptide have been documented in AN (Moriya et al., 2006), but it is so far unknown if this change remains after weight recovery. The changed cerebrospinal fluid levels of NPY seen in AN is however, known to be secondary to the illness (Gendall et al., 1999).

In addition, an increased expression of the neurotrophic receptor kinase 3 (*Ntrk3)* gene has been shown in the *anx/anx* hypothalamus (Mercader et al., 2008b). This agrees with AN being associated with specific variants of the genes for brain derived neurotrophic factor (BDNF) and neurotrophic receptor tyrosine kinase 2 (NTRK2) (Ribases et al., 2003, 2005).

Changes have been documented also in other brain regions than the hypothalamus. Increased apoptosis and proliferation in the dentate gyrus of the hippocampus (Kim et al., 2001), serotonergic hyperinnervation in hippocampus, cortex, olfactory bulb and cerebellum (Son et al., 1994), as well as altered dopaminergic transmission in the striatum (Johansen et al., 2001), have been demonstrated. Genetic variants as well as deviant levels of metabolites and receptors related to dopamine and serotonin have been linked to the AN pathology (Kaye et al., 1999, 2005; Kaye, 2008).

### Neuroinflammation and Degeneration in the anx/anx Hypothalamus

The hypothalamic neurochemical aberrances of the *anx/anx* mouse are accompanied by signs of inflammation and degeneration (Lachuer et al., 2005; Mercader et al., 2008a; Nilsson et al., 2008, 2011). Microglia cells are immunocompetent cells that are activated in the central nervous system in response to e.g., inflammation, neurodegeneration or injury (Nakajima and Kohsaka, 2004; Streit et al., 2005). In the *anx/anx* brain, microglia are activated selectively in the hypothalamic regions where the neurons, both cell bodies and projections, expressing the orexigenic neuropeptide AGRP are located (Nilsson et al., 2008). The first appearance of activated microglia overlaps in time with the loss of AGRP immunoreactive projections, i.e., P12–15 (Nilsson et al., 2008). Similarly, chemical ablation of the AGRP neurons results in starvation in both normal weight and obese mice, and results in glia (microglia and astroglia) activation in the target areas (Wu et al., 2008, 2012). Major histocompatibility complex I is expressed by the activated microglia, but also by the AGRP and POMC expressing neurons in the *anx/anx* brain (Nilsson et al., 2011). This latter finding combined with increased hypothalamic terminal dUTP nick end labeling (TUNEL) labeling and so called microglia-associated cell death (Ribak et al., 2009), made us conclude that hypothalamic degeneration is associated with the anorexia of the *anx/anx* mouse (Nilsson et al., 2011). In addition, two microarray studies of the *anx/anx* hypothalamus revealed changed expression of an enrichment of genes involved in inflammation and cell death (Lachuer et al., 2005; Mercader et al., 2008a). While it is unknown if hypothalamic inflammation occurs in AN, it has been linked to cachexia, the anorexia that often accompanies chronic illnesses such as cancer and HIV (Durham et al., 2009; Dwarkasing et al., 2016).

### Mitochondrial CI Dysfunction and Reduced Hypothalamic Metabolism

A dysfunction selective of CI in OXPHOS, and subsequent increased oxidative stress, have been revealed in the hypothalamus of the *anx/anx* mouse (Lindfors et al., 2011). This CI dysfunction is connected to down regulation of the gene *Ndufaf1* which in fact is located in the *anx* interval (see section on the *anx* mutation above). The down regulation has been confirmed at the protein level at P21 (Lindfors et al., 2011). *Ndufaf1* encodes one of several proteins crucial for the correct assembly of the 44–46 proteins that build up CI (Smeitink et al., 2001; Ugalde et al., 2004a,b; Guerrero-Castillo et al., 2017). Selective neuronal damage and glia activation, as shown in the *anx/anx* mouse (Nilsson et al., 2008, 2011), has been shown in another animal model with CI deficiencies, i.e., the Ndufs4-KO mouse (Quintana et al., 2010). The NDUFAF1 gene, as well as other players in CI biogenesis, have been implicated in human pathology; resulting in e.g., leukodystrophy and failure to thrive in young children (Vogel et al., 2005, 2007; Dunning et al., 2007; Distelmaier et al., 2009). In fact, CI dysfunction has been shown in leukocytes from patients with AN (Victor et al., 2014), but it remains to be explored if this is a cause or consequence of the disorder. This far, the NDUFAF1 gene has not been associated with AN, but it would be worth exploring genetics variants related to OXPHOS function and a potential association with AN, similar to what has been shown in other psychiatric disorders e.g., autism spectrum disorder (Giulivi et al., 2010). With this saying the *anx/anx* model is a model of value for research on all human conditions with loss of appetite i.e., anorexia, including the anorexia seen in cachexia and failure to thrive, as well as AN. The *anx/anx* mouse is unique in the sense that few other models exist were the mice, similarly to the human conditions just mentioned, eat insufficient despite having full access to food. This in contrast to models were the researcher in one way or another limits the access of food (Siegfried et al., 2003).

Diseases associated with mitochondrial dysfunction are commonly associated with a stressed metabolic profile, and hypermetabolism (Wredenberg et al., 2006; Jeppesen et al., 2007; Milone and Wong, 2013). Supposedly such metabolic responses occur in order to safeguard adequate levels of ATP. In some cases, conversely, mitochondrial dysfunction is associated with reduced glucose uptake and hypometabolism, e.g., in Alzheimer’s disease and epilepsy (Chandrasekaran et al., 1996; Tenney et al., 2014). This resembles what we saw in the *anx/anx* hypothalamus, i.e., lower glucose uptake rate, decreased lactate content, as well as elevated phosphocreatine (PCr) content and reduced activation of AMP-activated kinase (AMPK) in the basal state (Bergstrom et al., 2017). This is similar to the hypometabolic state seen in hibernation (Healy et al., 2011) and could be reflecting lower neuronal activity (Cunnane et al., 2011). Different neuronal populations respond differently to this type of metabolic stress (Schreiber and Baudry, 1995), which has been ascribed to the subtype of ATP-sensitive potassium channel (K-ATP) they express. A specific subtype of K-ATP channel that consists of Kir 6.2 and SUR1 subunits becomes activated by mitochondrial CI dysfunction, i.e., by increased ROS levels and/or reduced levels of ATP. This leads to ceased electrical activity, hyperpolarization and reduced firing, in a means of protecting the cell from the energy deficiency and increased oxidative stress (Liss et al., 1999). Kir6.2/SUR1 K-ATP channels are expressed by the hypothalamic POMC/CART and AGRP/NPY neurons, and by a limited number of other cell populations including the pancreatic beta-cells and dopaminergic neurons in Substantia Nigra (Miki et al., 2001; Ibrahim et al., 2003; van den Top and Spanswick, 2006; van den Top et al., 2007). Firing of action potentials and release of neurotransmitters are processes that require high amounts of energy. Therefore, inhibition of these processes would conserve energy during conditions when energy is scarce (Attwell and Laughlin, 2001; Sengupta et al., 2010). In addition, uncontrolled generation of ROS, commonly accompanying CI dysfunction, can also cause diminished firing of the AGRP/NPY neurons, thus resulting in a reduced orexigenic drive (Andrews et al., 2008; Horvath et al., 2009).

### Pancreatic Dysfunction and Aberrant Levels of Fat Derived Molecules

The *anx/anx* mouse also displays a pancreatic dysfunction (Lindfors et al., 2015). More specifically, they are markedly glucose intolerant, and show reduced insulin release upon glucose tolerance test. This is associated with elevated serum concentrations of free fatty acids (FFAs) in the *anx/anx* mouse and increased macrophage infiltration [indicative of inflammation (Imai et al., 1996; Ka et al., 2015)] of *anx/anx* islets. Increased levels of FFAs have been connected to inhibition of glucose-induced insulin secretion (Eguchi et al., 2012). Interestingly, isolated *anx/anx* islets cultured in the absence of FFAs show increased insulin release upon glucose stimulation and show no signs of inflammation. Thus, the diabetic phenotype of the *anx/anx* mouse seems to be related to the elevated FFAs and inflammation in pancreatic islets. This finding is interesting in the light of the increased incidence of eating disorders that has been reported in young women with diabetes (Hudson et al., 1985; Meltzer et al., 2001), and documented increased levels of circulating FFAs in AN (Pinter et al., 1975; Curatola et al., 2004). Also similar to individuals with AN, the *anx/anx* mouse has low levels of the fat derived and food intake regulating hormone leptin, and high levels of cholesterol in serum (Maltais et al., 1984; Schorr and Miller, 2017).

## Conclusion and Future Perspective

The *anx/anx* mouse shares several characteristics with patients with AN, including emaciation, starvation, premature death, diabetic features, increased FFA and low leptin, and is therefore a unique and very valuable resource in order to explore and understand the (neuro)biology of AN. Future research should explore if hypothalamic inflammation and/or degeneration, as seen in the *anx/anx* mouse, are mechanisms involved also in AN. Further studies are also needed in order to clarify if the mitochondrial dysfunction seen in AN (Victor et al., 2014) is a cause or consequence of the disorder. Finally, it would be of value to be able to define the *anx*-mutation, as well as explore other brain areas related to food intake regulation, e.g., nucleus tractus solitarius and the parabrachial nucleus in the *anx/anx* mouse.

## Author Contributions

IN reviewed the literature, wrote, and edited the manuscript.

## Conflict of Interest Statement

The author declares that the research was conducted in the absence of any commercial or financial relationships that could be construed as a potential conflict of interest.

## Footnotes

1 www.ensembl.org

## References

[B1] Andrews Z. B. Liu Z. W. Walllingford N. Erion D. M. Borok E. Friedman J. M. (2008). UCP2 mediates ghrelin’s action on NPY/AgRP neurons by lowering free radicals. *Nature* 454 846–851. 10.1038/nature07181 18668043 PMC4101536

[B2] Attwell D. Laughlin S. B. (2001). An energy budget for signaling in the grey matter of the brain. *J. Cereb. Blood Flow Metab.* 21 1133–1145. 10.1097/00004647-200110000-00001 11598490

[B3] Bergstrom U. Lindfors C. Svedberg M. Johansen J. E. Haggkvist J. Schalling M. (2017). Reduced metabolism in the hypothalamus of the anorectic anx/anx mouse. *J. Endocrinol.* 233 15–24. 10.1530/JOE-16-0383 28130409

[B4] Broberger C. Johansen J. Brismar H. Johansson C. Schalling M. Hokfelt T. (1999). Changes in neuropeptide Y receptors and pro-opiomelanocortin in the anorexia (anx/anx) mouse hypothalamus. *J. Neurosci.* 19 7130–7139. 10.1523/JNEUROSCI.19-16-07130.1999 10436066 PMC6782872

[B5] Broberger C. Johansen J. Johansson C. Schalling M. Hokfelt T. (1998). The neuropeptide Y/agouti gene-related protein (AGRP) brain circuitry in normal, anorectic, and monosodium glutamate-treated mice. *Proc. Natl. Acad. Sci. U.S.A.* 95 15043–15048. 10.1073/pnas.95.25.15043 9844012 PMC24572

[B6] Broberger C. Johansen J. Schalling M. Hokfelt T. (1997). Hypothalamic neurohistochemistry of the murine anorexia (anx/anx) mutation: altered processing of neuropeptide Y in the arcuate nucleus. *J. Comp. Neurol.* 387 124–135. 10.1002/(SICI)1096-9861(19971013)387:1<124::AID-CNE10>3.0.CO;2-U 9331176

[B7] Bulik C. M. Sullivan P. F. Tozzi F. Furberg H. Lichtenstein P. Pedersen N. L. (2006). Prevalence, heritability, and prospective risk factors for anorexia nervosa. *Arch. Gen. Psychiatry* 63 305–312. 10.1001/archpsyc.63.3.305 16520436

[B8] Chandrasekaran K. Hatanpaa K. Brady D. R. Rapoport S. I. (1996). Evidence for physiological down-regulation of brain oxidative phosphorylation in Alzheimer’s disease. *Exp. Neurol.* 142 80–88. 10.1006/exnr.1996.0180 8912900

[B9] Chronwall B. M. (1985). Anatomy and physiology of the neuroendocrine arcuate nucleus. *Peptides* 6(Suppl. 2) 1–11. 10.1016/0196-9781(85)90128-7 2417205

[B10] Cone R. D. Cowley M. A. Butler A. A. Fan W. Marks D. L. Low M. J. (2001). The arcuate nucleus as a conduit for diverse signals relevant to energy homeostasis. *Int. J. Obes. Relat. Metab. Disord.* 25(Suppl. 5) S63–S67. 10.1038/sj.ijo.0801913 11840218

[B11] Cunnane S. Nugent S. Roy M. Courchesne-Loyer A. Croteau E. Tremblay S. (2011). Brain fuel metabolism, aging, and Alzheimer’s disease. *Nutrition* 27 3–20. 10.1016/j.nut.2010.07.021 21035308 PMC3478067

[B12] Curatola G. Camilloni M. A. Vignini A. Nanetti L. Boscaro M. Mazzanti L. (2004). Chemical-physical properties of lipoproteins in anorexia nervosa. *Eur. J. Clin. Invest.* 34 747–751. 10.1111/j.1365-2362.2004.01415.x 15530147

[B13] Dardennes R. M. Zizzari P. Tolle V. Foulon C. Kipman A. Romo L. (2007). Family trios analysis of common polymorphisms in the obestatin/ghrelin, BDNF and AGRP genes in patients with Anorexia nervosa: association with subtype, body-mass index, severity and age of onset. *Psychoneuroendocrinology* 32 106–113. 10.1016/j.psyneuen.2006.11.003 17197106

[B14] DeBoer M. D. (2011). What can anorexia nervosa teach us about appetite regulation? *Nutrition* 27 405–406. 10.1016/j.nut.2011.02.001 21392703 PMC3062266

[B15] Distelmaier F. Koopman W. J. van den Heuvel L. P. Rodenburg R. J. Mayatepek E. Willems P. H. (2009). Mitochondrial complex I deficiency: from organelle dysfunction to clinical disease. *Brain* 132(Pt 4) 833–842. 10.1093/brain/awp058 19336460

[B16] Duncan L. Yilmaz Z. Gaspar H. Walters R. Goldstein J. Anttila V. (2017). Significant locus and metabolic genetic correlations revealed in genome-wide association study of anorexia nervosa. *Am. J. Psychiatry* 174 850–858. 10.1176/appi.ajp.2017.16121402 28494655 PMC5581217

[B17] Dunning C. J. McKenzie M. Sugiana C. Lazarou M. Silke J. Connelly A. (2007). Human CIA30 is involved in the early assembly of mitochondrial complex I and mutations in its gene cause disease. *EMBO J.* 26 3227–3237. 10.1038/sj.emboj.7601748 17557076 PMC1914096

[B18] Durham W. J. Dillon E. L. Sheffield-Moore M. (2009). Inflammatory burden and amino acid metabolism in cancer cachexia. *Curr. Opin. Clin. Nutr. Metab. Care* 12 72–77. 10.1097/MCO.0b013e32831cef61 19057191 PMC2742684

[B19] Dwarkasing J. T. Marks D. L. Witkamp R. F. van Norren K. (2016). Hypothalamic inflammation and food intake regulation during chronic illness. *Peptides* 77 60–66. 10.1016/j.peptides.2015.06.011 26158772

[B20] Eguchi K. Manabe I. Oishi-Tanaka Y. Ohsugi M. Kono N. Ogata F. (2012). Saturated fatty acid and TLR signaling link beta cell dysfunction and islet inflammation. *Cell Metab.* 15 518–533. 10.1016/j.cmet.2012.01.023 22465073

[B21] Fetissov S. O. Bergstrom U. Johansen J. E. Hokfelt T. Schalling M. Ranscht B. (2005). Alterations of arcuate nucleus neuropeptidergic development in contactin-deficient mice: comparison with anorexia and food-deprived mice. *Eur. J. Neurosci.* 22 3217–3228. 10.1111/j.1460-9568.2005.04513.x 16367788

[B22] Gendall K. A. Kaye W. H. Altemus M. McConaha C. W. La Via M. C. (1999). Leptin, neuropeptide Y, and peptide YY in long-term recovered eating disorder patients. *Biol. Psychiatry* 46 292–299. 10.1016/S0006-3223(98)00292-3 10418705

[B23] Giulivi C. Zhang Y. F. Omanska-Klusek A. Ross-Inta C. Wong S. Hertz-Picciotto I. (2010). Mitochondrial dysfunction in autism. *JAMA* 304 2389–2396. 10.1001/jama.2010.1706 21119085 PMC3915058

[B24] Guerrero-Castillo S. Baertling F. Kownatzki D. Wessels H. J. Arnold S. Brandt U. (2017). The assembly pathway of mitochondrial respiratory chain complex I. *Cell Metab.* 25 128–139. 10.1016/j.cmet.2016.09.002 27720676

[B25] Healy J. E. Gearhart C. N. Bateman J. L. Handa R. J. Florant G. L. (2011). AMPK and ACCchange with fasting and physiological condition in euthermic and hibernating golden-mantled ground squirrels (*Callospermophilus lateralis*). *Comp. Biochem. Physiol. A Mol. Integr. Physiol.* 159 322–331. 10.1016/j.cbpa.2011.03.026 21473923 PMC3090470

[B26] Hinney A. Kesselmeier M. Jall S. Volckmar A. L. Focker M. Antel J. (2017). Evidence for three genetic loci involved in both anorexia nervosa risk and variation of body mass index. *Mol. Psychiatry* 22 321–322. 10.1038/mp.2016.126 27457816 PMC8477229

[B27] Horvath T. L. Andrews Z. B. Diano S. (2009). Fuel utilization by hypothalamic neurons: roles for ROS. *Trends Endocrinol. Metab.* 20 78–87. 10.1016/j.tem.2008.10.003 19084428

[B28] Hudson J. I. Wentworth S. M. Hudson M. S. Pope H. G. Jr (1985). Prevalence of anorexia nervosa and bulimia among young diabetic women. *J. Clin. Psychiatry* 46 88–89. 3871764

[B29] Ibrahim N. Bosch M. A. Smart J. L. Qiu J. Rubinstein M. Ronnekleiv O. K. (2003). Hypothalamic proopiomelanocortin neurons are glucose responsive and express K(ATP) channels. *Endocrinology* 144 1331–1340. 10.1210/en.2002-221033 12639916

[B30] Imai Y. Ibata I. Ito D. Ohsawa K. Kohsaka S. (1996). A novel gene iba1 in the major histocompatibility complex class III region encoding an EF hand protein expressed in a monocytic lineage. *Biochem. Biophys. Res. Commun.* 224 855–862. 10.1006/bbrc.1996.1112 8713135

[B31] Jahng J. W. Houpt T. A. Kim S. J. Joh T. H. Son J. H. (1998). Neuropeptide Y mRNA and serotonin innervation in the arcuate nucleus of anorexia mutant mice. *Brain Res.* 790 67–73. 10.1016/S0006-8993(98)00049-3 9593828

[B32] Jeppesen T. D. Quistorff B. Wibrand F. Vissing J. (2007). 31P-MRS of skeletal muscle is not a sensitive diagnostic test for mitochondrial myopathy. *J. Neurol.* 254 29–37. 10.1007/s00415-006-0229-5 17278044

[B33] Johansen J. E. Broberger C. Lavebratt C. Johansson C. Kuhar M. J. Hokfelt T. (2000). Hypothalamic CART and serum leptin levels are reduced in the anorectic (anx/anx) mouse. *Brain Res. Mol. Brain Res.* 84 97–105. 10.1016/S0169-328X(00)00228-X 11113536

[B34] Johansen J. E. Fetissov S. Fischer H. Arvidsson S. Hokfelt T. Schalling M. (2003). Approaches to anorexia in rodents: focus on the anx/anx mouse. *Eur. J. Pharmacol.* 480 171–176. 10.1016/j.ejphar.2003.08.104 14623360

[B35] Johansen J. E. Teixeira V. L. Johansson C. Serrao P. Berggren P. O. Soares-Da-Silva P. (2001). Altered dopaminergic transmission in the anorexic anx/anx mouse striatum. *Neuroreport* 12 2737–2741. 10.1097/00001756-200108280-00029 11522958

[B36] Ka S. O. Song M. Y. Bae E. J. Park B. H. (2015). Myeloid SIRT1 regulates macrophage infiltration and insulin sensitivity in mice fed a high-fat diet. *J. Endocrinol.* 224 109–118. 10.1530/JOE-14-0527 25349250

[B37] Kaye W. (2008). Neurobiology of anorexia and bulimia nervosa. *Physiol. Behav.* 94 121–135. 10.1016/j.physbeh.2007.11.037 18164737 PMC2601682

[B38] Kaye W. H. Frank G. K. Bailer U. F. Henry S. E. Meltzer C. C. Price J. C. (2005). Serotonin alterations in anorexia and bulimia nervosa: new insights from imaging studies. *Physiol. Behav.* 85 73–81. 10.1016/j.physbeh.2005.04.013 15869768

[B39] Kaye W. H. Frank G. K. McConaha C. (1999). Altered dopamine activity after recovery from restricting-type anorexia nervosa. *Neuropsychopharmacology* 21 503–506. 10.1016/S0893-133X(99)00053-6 10481833

[B40] Keski-Rahkonen A. Hoek H. W. Susser E. S. Linna M. S. Sihvola E. Raevuori A. (2007). Epidemiology and course of anorexia nervosa in the community. *Am. J. Psychiatry* 164 1259–1265. 10.1176/appi.ajp.2007.06081388 17671290

[B41] Kim D. Y. Yu J. Mui R. K. Niibori R. Taufique H. B. Aslam R. (2017). The tyrosine kinase receptor Tyro3 enhances lifespan and neuropeptide Y (Npy) neuron survival in the mouse anorexia (anx) mutation. *Dis. Model. Mech.* 10 581–595. 10.1242/dmm.027433 28093506 PMC5451163

[B42] Kim M. J. Kim Y. Kim S. A. Lee H. J. Choe B. K. Nam M. (2001). Increases in cell proliferation and apoptosis in dentate gyrus of anorexia (anx/anx) mice. *Neurosci. Lett.* 302 109–112. 10.1016/S0304-3940(01)01684-6 11290399

[B43] Kopp J. Xu Z. Q. Zhang X. Pedrazzini T. Herzog H. Kresse A. (2002). Expression of the neuropeptide Y Y1 receptor in the CNS of rat and of wild-type and Y1 receptor knock-out mice. Focus on immunohistochemical localization. *Neuroscience* 111 443–532. 10.1016/S0306-4522(01)00463-8 12031341

[B44] Lachuer J. Ouyang L. Legras C. Del Rio J. Barlow C. (2005). Gene expression profiling reveals an inflammatory process in the anx/anx mutant mice. *Brain Res. Mol. Brain Res.* 139 372–376. 10.1016/j.molbrainres.2005.06.003 16006007

[B45] Lindfors C. Katz A. Selander L. Johansen J. E. Marconi G. Schalling M. (2015). Glucose intolerance and pancreatic beta-cell dysfunction in the anorectic anx/anx mouse. *Am. J. Physiol. Endocrinol. Metab.* 309 E418–E427. 10.1152/ajpendo.00081.2015 26126683

[B46] Lindfors C. Nilsson I. A. Garcia-Roves P. M. Zuberi A. R. Karimi M. Donahue L. R. (2011). Hypothalamic mitochondrial dysfunction associated with anorexia in the anx/anx mouse. *Proc. Natl. Acad. Sci. U.S.A.* 108 18108–18113. 10.1073/pnas.1114863108 22025706 PMC3207677

[B47] Liss B. Bruns R. Roeper J. (1999). Alternative sulfonylurea receptor expression defines metabolic sensitivity of K-ATP channels in dopaminergic midbrain neurons. *EMBO J.* 18 833–846. 10.1093/emboj/18.4.833 10022826 PMC1171176

[B48] Maltais L. J. Lane P. W. Beamer W. G. (1984). Anorexia, a recessive mutation causing starvation in preweanling mice. *J. Hered.* 75 468–472. 10.1093/oxfordjournals.jhered.a109987 6595305

[B49] Meltzer L. J. Johnson S. B. Prine J. M. Banks R. A. Desrosiers P. M. Silverstein J. H. (2001). Disordered eating, body mass, and glycemic control in adolescents with type 1 diabetes. *Diabetes Care* 24 678–682. 10.2337/diacare.24.4.678 11315830

[B50] Mercader J. M. Lozano J. J. Sumoy L. Dierssen M. Visa J. Gratacos M. (2008a). Hypothalamus transcriptome profile suggests an anorexia-cachexia syndrome in the anx/anx mouse model. *Physiol. Genom.* 35 341–350. 10.1152/physiolgenomics.90255.2008 18812457

[B51] Mercader J. M. Saus E. Aguera Z. Bayes M. Boni C. Carreras A. (2008b). Association of NTRK3 and its interaction with NGF suggest an altered cross-regulation of the neurotrophin signaling pathway in eating disorders. *Hum. Mol. Genet.* 17 1234–1244. 10.1093/hmg/ddn013 18203754

[B52] Miki T. Liss B. Minami K. Shiuchi T. Saraya A. Kashima Y. (2001). ATP-sensitive K+ channels in the hypothalamus are essential for the maintenance of glucose homeostasis. *Nat. Neurosci.* 4 507–512. 10.1038/87455 11319559

[B53] Milone M. Wong L. J. (2013). Diagnosis of mitochondrial myopathies. *Mol. Genet. Metab.* 110 35–41. 10.1016/j.ymgme.2013.07.007 23911206

[B54] Moriya J. Takimoto Y. Yoshiuchi K. Shimosawa T. Akabayashi A. (2006). Plasma agouti-related protein levels in women with anorexia nervosa. *Psychoneuroendocrinology* 31 1057–1061. 10.1016/j.psyneuen.2006.06.006 16904835

[B55] Nakajima K. Kohsaka S. (2004). Microglia: neuroprotective and neurotrophic cells in the central nervous system. *Curr. Drug Targets Cardiovasc. Haematol. Disord.* 4 65–84. 15032653 10.2174/1568006043481284

[B56] Nilsson I. Lindfors C. Fetissov S. O. Hokfelt T. Johansen J. E. (2008). Aberrant agouti-related protein system in the hypothalamus of the anx/anx mouse is associated with activation of microglia. *J. Comp. Neurol.* 507 1128–1140. 10.1002/cne.21599 18098136

[B57] Nilsson I. A. Lindfors C. Schalling M. Hokfelt T. Johansen J. E. (2013). Anorexia and hypothalamic degeneration. *Vitam. Horm.* 92 27–60. 10.1016/B978-0-12-410473-0.00002-7 23601420

[B58] Nilsson I. A. Thams S. Lindfors C. Bergstrand A. Cullheim S. Hokfelt T. (2011). Evidence of hypothalamic degeneration in the anorectic anx/anx mouse. *Glia* 59 45–57. 10.1002/glia.21075 20967882

[B59] Oberndorfer T. A. Frank G. K. Simmons A. N. Wagner A. McCurdy D. Fudge J. L. (2013). Altered insula response to sweet taste processing after recovery from anorexia and bulimia nervosa. *Am. J. Psychiatry* 170 1143–1151. 10.1176/appi.ajp.2013.11111745 23732817 PMC3971875

[B60] Papadopoulos F. C. Ekbom A. Brandt L. Ekselius L. (2009). Excess mortality, causes of death and prognostic factors in anorexia nervosa. *Br. J. Psychiatry* 194 10–17. 10.1192/bjp.bp.108.054742 19118319

[B61] Pietilainen K. H. Saarni S. E. Kaprio J. Rissanen A. (2012). Does dieting make you fat? A twin study. *Int. J. Obes.* 36 456–464. 10.1038/ijo.2011.160 21829159

[B62] Pinter E. J. Tolis G. Friesen H. G. (1975). L-dopa, growth hormone and adipokinesis in the lean and the obese. *Int. J. Clin. Pharmacol. Biopharm.* 12 277–280. 1165135

[B63] Quintana A. Kruse S. E. Kapur R. P. Sanz E. Palmiter R. D. (2010). Complex I deficiency due to loss of Ndufs4 in the brain results in progressive encephalopathy resembling Leigh syndrome. *Proc. Natl. Acad. Sci. U.S.A.* 107 10996–11001. 10.1073/pnas.1006214107 20534480 PMC2890717

[B64] Ribak C. E. Shapiro L. A. Perez Z. D. Spigelman I. (2009). Microglia-associated granule cell death in the normal adult dentate gyrus. *Brain Struct. Funct.* 214 25–35. 10.1007/s00429-009-0231-7 19936784 PMC2782120

[B65] Ribases M. Gratacos M. Armengol L. de Cid R. Badia A. Jimenez L. (2003). Met66 in the brain-derived neurotrophic factor (BDNF) precursor is associated with anorexia nervosa restrictive type. *Mol. Psychiatry* 8 745–751. 10.1038/sj.mp.4001281 12888803

[B66] Ribases M. Gratacos M. Badia A. Jimenez L. Solano R. Vallejo J. (2005). Contribution of NTRK2 to the genetic susceptibility to anorexia nervosa, harm avoidance and minimum body mass index. *Mol. Psychiatry* 10 851–860. 10.1038/sj.mp.4001670 15838534

[B67] Schaumberg K. Welch E. Breithaupt L. Hubel C. Baker J. H. Munn-Chernoff M. A. (2017). The science behind the academy for eating disorders’ nine truths about eating disorders. *Eur. Eat. Disord. Rev.* 25 432–450. 10.1002/erv.2553 28967161 PMC5711426

[B68] Schorr M. Miller K. K. (2017). The endocrine manifestations of anorexia nervosa: mechanisms and management. *Nat. Rev. Endocrinol.* 13 174–186. 10.1038/nrendo.2016.175 27811940 PMC5998335

[B69] Schreiber S. S. Baudry M. (1995). Selective neuronal vulnerability in the hippocampus–a role for gene expression? *Trends Neurosci.* 18 446–451. 8545911 10.1016/0166-2236(95)94495-q

[B70] Schwartz M. W. (2001). Brain pathways controlling food intake and body weight. *Exp. Biol. Med.* 226 978–981. 10.1177/153537020122601103 11743132

[B71] Sengupta B. Stemmler M. Laughlin S. B. Niven J. E. (2010). Action potential energy efficiency varies among neuron types in vertebrates and invertebrates. *PLoS Comput. Biol.* 6:e1000840. 10.1371/journal.pcbi.1000840 20617202 PMC2895638

[B72] Siegfried Z. Berry E. M. Hao S. Avraham Y. (2003). Animal models in the investigation of anorexia. *Physiol. Behav.* 79 39–45. 10.1016/S0031-9384(03)00103-3 12818708

[B73] Smeitink J. van den Heuvel L. DiMauro S. (2001). The genetics and pathology of oxidative phosphorylation. *Nat. Rev. Genet.* 2 342–352. 10.1038/35072063 11331900

[B74] Son J. H. Baker H. Park D. H. Joh T. H. (1994). Drastic and selective hyperinnervation of central serotonergic neurons in a lethal neurodevelopmental mouse mutant, Anorexia (anx). *Brain Res. Mol. Brain Res.* 25 129–134. 10.1016/0169-328X(94)90287-9 7984037

[B75] Streit W. J. Conde J. R. Fendrick S. E. Flanary B. E. Mariani C. L. (2005). Role of microglia in the central nervous system’s immune response. *Neurol. Res.* 27 685–691. 10.1179/016164105X49463 16197805

[B76] Tenney J. R. Rozhkov L. Horn P. Miles L. Miles M. V. (2014). Cerebral glucose hypometabolism is associated with mitochondrial dysfunction in patients with intractable epilepsy and cortical dysplasia. *Epilepsia* 55 1415–1422. 10.1111/epi.12731 25053176

[B77] Ugalde C. Janssen R. J. van den Heuvel L. P. Smeitink J. A. Nijtmans L. G. (2004a). Differences in assembly or stability of complex I and other mitochondrial OXPHOS complexes in inherited complex I deficiency. *Hum. Mol. Genet.* 13 659–667. 10.1093/hmg/ddh071 14749350

[B78] Ugalde C. Vogel R. Huijbens R. Van Den Heuvel B. Smeitink J. Nijtmans L. (2004b). Human mitochondrial complex I assembles through the combination of evolutionary conserved modules: a framework to interpret complex I deficiencies. *Hum. Mol. Genet.* 13 2461–2472. 15317750 10.1093/hmg/ddh262

[B79] van den Top M. Lyons D. J. Lee K. Coderre E. Renaud L. P. Spanswick D. (2007). Pharmacological and molecular characterization of ATP-sensitive K(+) conductances in CART and NPY/AgRP expressing neurons of the hypothalamic arcuate nucleus. *Neuroscience* 144 815–824. 10.1016/j.neuroscience.2006.09.059 17137725

[B80] van den Top M. Spanswick D. (2006). Integration of metabolic stimuli in the hypothalamic arcuate nucleus. *Prog. Brain Res.* 153 141–154. 10.1016/S0079-6123(06)53008-0 16876573

[B81] Victor V. M. Rovira-Llopis S. Saiz-Alarcon V. Sanguesa M. C. Rojo-Bofill L. Banuls C. (2014). Altered mitochondrial function and oxidative stress in leukocytes of anorexia nervosa patients. *PLoS One* 9:e106463. 10.1371/journal.pone.0106463 25254642 PMC4177818

[B82] Vogel R. O. Janssen R. J. Ugalde C. Grovenstein M. Huijbens R. J. Visch H. J. (2005). Human mitochondrial complex I assembly is mediated by NDUFAF1. *FEBS J.* 272 5317–5326. 10.1111/j.1742-4658.2005.04928.x 16218961

[B83] Vogel R. O. Janssen R. J. van den Brand M. A. Dieteren C. E. Verkaart S. Koopman W. J. (2007). Cytosolic signaling protein Ecsit also localizes to mitochondria where it interacts with chaperone NDUFAF1 and functions in complex I assembly. *Genes Dev.* 21 615–624. 10.1101/gad.408407 17344420 PMC1820902

[B84] Wredenberg A. Freyer C. Sandstrom M. E. Katz A. Wibom R. Westerblad H. (2006). Respiratory chain dysfunction in skeletal muscle does not cause insulin resistance. *Biochem. Biophys. Res. Commun.* 350 202–207. 10.1016/j.bbrc.2006.09.029 16996481

[B85] Wu Q. Howell M. P. Palmiter R. D. (2008). Ablation of neurons expressing agouti-related protein activates fos and gliosis in postsynaptic target regions. *J. Neurosci.* 28 9218–9226. 10.1523/JNEUROSCI.2449-08.2008 18784302 PMC2597113

[B86] Wu Q. Whiddon B. B. Palmiter R. D. (2012). Ablation of neurons expressing agouti-related protein, but not melanin concentrating hormone, in leptin-deficient mice restores metabolic functions and fertility. *Proc. Natl. Acad. Sci. U.S.A.* 109 3155–3160. 10.1073/pnas.1120501109 22232663 PMC3286929

[B87] Yilmaz Z. Kaplan A. S. Tiwari A. K. Levitan R. D. Piran S. Bergen A. W. (2014). The role of leptin, melanocortin, and neurotrophin system genes on body weight in anorexia nervosa and bulimia nervosa. *J. Psychiatr. Res.* 55 77–86. 10.1016/j.jpsychires.2014.04.005 24831852 PMC4191922

[B88] Zhang X. Bao L. Xu Z. Q. Kopp J. Arvidsson U. Elde R. (1994). Localization of neuropeptide Y Y1 receptors in the rat nervous system with special reference to somatic receptors on small dorsal root ganglion neurons. *Proc. Natl. Acad. Sci. U.S.A.* 91 11738–11742. 10.1073/pnas.91.24.11738 7972133 PMC45307

